# Reliability and validity of measuring scale for postoperative complications in third molar surgery

**DOI:** 10.1186/s12903-018-0486-6

**Published:** 2018-02-21

**Authors:** Pedro Christian Aravena, Paula Astudillo, Horacio Miranda, Carlos Manterola

**Affiliations:** 1 Dental School, Faculty of Medicine, Universidad Austral de Chile, Valdivia, Chile; 2 Program in PhD Medical Science, Department of Surgery and Traumatology, Faculty of Medicine, Universidad de La Frontera, Temuco, Chile; 3 Program in Psychology, Health and Quality of Life, Universidad de Girona, Girona, Spain; 4 Faculty of Forest and Agropecuary Science, Universidad de La Frontera, Temuco, Chile; 5 Center of Biomedical Research, Universidad Autónoma de Chile, Providencia, Chile; 6 Faculty of Dentistry, Universidad San Sebastián, Santiago, Chile

**Keywords:** Third molar, Postoperative complications, Measurement scale, factor analysis, Oral surgery

## Abstract

**Background:**

Third molar removal surgery is the most frequently performed surgery in the oral and maxillofacial field with a wide range of items in the quantification of postoperative complications. For their measure, in 2014 a previous scale design was presented. The aim of this study was to determine the reliability and validity of a scale designed to measure and quantify postoperative complications in third molar surgery (TMS).

**Methods:**

A cross-sectional study of a measurement model was designed. Sixty-two patients (mean age 20.5 ± 6.6 years; 36 women) underwent TMS in three Chilean hospitals. In the postoperative check-up on the 7th day, a maxillofacial surgeon and a surgical resident performed independent postoperative assessments, applying the scale*.* A confirmatory factor analysis was conducted to obtain validity, internal consistency, interobserver reliability and a score to categorize the severity of complications using structural equation model analysis.

**Results:**

Nine patients (14.5%) had complications. The scale was defined by two components: “Secondary complication” and “Infection” (Cronbach’s alpha 0.71; Interobserver reliability 87.7%) and three categories of postoperative complication: “without or mild”, “moderate” and “severe”.

**Conclusion:**

This study presents a reliability and validity scale called “Surgical complication assessment scale in TMS”.

## Background

Third molar removal surgery (TMS) is the most frequently performed surgery in the oral and maxillofacial field [1]. There is a wide range of items in the quantification of post-operative complications [2], which can appear in up to 75% of cases [3]. This great variability is due to the various factors: inconsistency in the diagnostic criteria and assessment methods used in the different studies, variation in surgical technique, the surgeon’s ability and experience, patient variability and the absence of valid and reliable measurement instruments to record objective and subjective postoperative complication variables [4–7].

Many measurement instruments in health lack a systematic report of their psychometric properties. It is recommended that at least two types of reliability, a content validity, construct validity or a criterion be presented in their publication [8]. An assessment scale of surgical complications in TMS was designed in 2014. It has three components and eight items (Table 1) and generates a construct that quantifies the degree of severity of postoperative complications in TMS [9]. The validity and reliability proprieties of this scale must be analyzed in order to contribute a new instrument that facilitates its clinical use and benefits the rapid classification of clinical status.

**Table 1:** Sociodemographic characteristics of the study participants

		Hospital		
Item		Santiago (*n* = 20)	Valdivia (*n* = 25)	Talcahuano (*n* = 17)
Age in years	Mean (SD)	24.7 (9.9)	17.7 (4.3)	20.2 (4.2)
Female		11	14	11
Level education	Primary	4	5	2
	Secondary	11	15	15
	Technical	0	2	1
	Professional	2	3	2
Smoker	None	12	20	17
	< 5 per day	3	4	3
	5–10 per day	1	1	0
Illness	None	17	23	14
	Hypertension	2	2	3
	Hypothyroidism	1	0	0

The aim of this study was to determine the validity and reliability of the previously designed scale in the clinical quantification of postoperative complications in patients who have had a TMS.

## Methods

A cross-sectional study with a measurement model was used. The psychometric properties of the previously designed instrument were assessed [9], estimating the validity of the scale according to its construct, criterion, discriminant validity and reliability by calculating the internal consistency, reproducibility and ease of interpretation of the results [10].

### Patients and observers

Sixty-two patients (36 women) from three Chilean hospitals (Hospital de la Base de Valdivia in Valdivia city, Hospital Higueras in Talcahuano city and the Hospital San Borja Arriarán in Santiago of Chile) with an average age of 20.5 ± 6.6 years were selected between April and October 2014 (Table 1). The number of patients was based on the scale design recommendations, including 10 respondents per item of the final scale [10]. The selection criteria were patients with an indication of TMS, ASA I, lucid when the informed consent was signed and who had completed the follow-up and postoperative check. Patients with a history of pericoronitis a month prior to surgery, treatment with antibiotics, steroidal or non-steroidal anti-inflammatories, allergies to derivatives of penicillin or corticosteroids, or who were pregnant were excluded.

In light of potential sources of bias according to the variability of surgical techniques and subjective evaluation that could be encountered during the evaluation of postoperative complications in TMS, maxillofacial surgeons (MFS) with more than 15 years of experience and working full-time in health centers were selected. In turn, observations of the postoperative complications were made by surgical residents with more than 5 years in maxillofacial surgery programs. Thus, seven MFS (53.7 ± 4.3 years of age with 23.6 ± 7.2 years of experience) and 12 surgical residents (28.8 ± 6.1 years of age and 5.21 ± 5.5 years of training) were observers randomized into two groups who applied the scale to the postoperative check-ups scheduled for the 7th day after TMS.

### Use of the scale

Each item of the previously designed scale with three components and eight items was assigned a measurement range from 0 to 10 according to what was recorded in previous reports [7, 10–13] and qualitative classifications provided by a group of experts who blended the operational definitions; therefore, the structure design (measurement model) is the stage prior to the present report [9] (Table 2).

**Table 2:** Scale design and range of postoperative complications applied in checks of patients after third molar surgery

Category	Item	Clinical picture	Scores
Secondary complication	Inflammation	None	1
		Mild (intraoral and surgical area)	3
		Moderate (intraoral and extraoral)	5
		Severe (intraoral, extraoral and other regions of the head)	10
	Erythema	None	1
		Intraoral	2
		Intraoral and extraoral	3
	Edema	None	1
		Intraoral	3
		Intraoral and extraoral	5
	Pain	How much pain does the patient feel at this moment?	0 to 10
	Trismus	Can the patient open his mouth without limitations?	0 to 5
Soft tissue infection	Abscess	Absent	1
		Present	5
	Suppuration	Absent	1
		Present when tissue is compressed	5
		Present spontaneously	10
Hard tissue infection	Alveolar osteitis	Absent	1
		Present	10

In each hospital, the principal investigator (PCA) explained the instructions of both the TMS protocol as well as the use of the scale. The MFS agreed to hold the surgical technique according to the diagnosis and third molar position [14]. Before all extractions, patients were given standard postoperative instructions. Initially, extraoral antisepsis was performed with 2% chlorhexidine gluconate and intraoral antisepsis with 0.12% chlorhexidine gluconate mouthwash for one minute. Anesthesia was done by a standard inferior alveolar nerve block and buccal blocks with lidocaine 1:100,000 (Xylonor 2%, Septodont®). A triangular mucoperiosteal flap was designed with an incision from the anterior border of the mandibular ramus to the distal surface of the distobuccal cusp of the mandibular second molar with a relieving vertical incision. Buccal osteotomy and tooth sectioning were carried out when necessary with a round bur under copious irrigation with 0.9% sterile saline, following the extraction performed. The flap was closed with interrupted 4–0 silk suture for the primary closure. After surgery, the following postoperative medication was prescribed: an antibiotic of amoxicillin 500 mg every eight hours for 5 days and 400 mg ibuprofen sodium taken orally every eight hours for pain relief.

For each patient, the MFS recorded the age (years), gender (male/female) and degree of surgical complexity (use of forceps/osteotomy/osteotomy and odontosection). On the 7th day of postoperative monitoring, a MFS and a surgical resident made independent postoperative assessments on the patients after verbal and written instructions regarding application of the scale. Both evaluated the presence of each clinical status according to the categories and items on the scale and assigned a score (Table 2). In addition, each wrote down his diagnostic impression of the clinical status, indicating the level of severity and clinical diagnosis (Table 3). This variability of visual inspections between the two observers (MFS or surgical resident) is recommended for the possibility of discrimination between different sorting options complications postsurgical the final scale [15].

**Table 3:** Level of severity recorded by the observers in the postoperative examination of patients after third molar surgery

Type of complication	Definition
No complication	No functional limitation or discomfort
Mild	Involvement of surgical wound
Moderate	Involvement of surgical wound and deep regions
Severe	Involvement of wound, deep regions and general condition

### Data analysis

A confirmatory factor analysis was performed by a second researcher (HM) to confirm the model of the previously designed scale [9]. This required 3 analytical stages:

#### Identification of outlier responses

In the matrix of collected data, outliers were identified and eliminated. These had been generated by tabulation errors or confirmed according to random distortion, such as reduced covariances and the correlations between the items on the scale [16].

#### Assessment of the scale model

A maximum likelihood confirmatory factor analysis (CFA) by means of a structural equations analysis was performed using the proposed measurement model evaluated with chi-square index (χ2). The linear structural relations model (LISREL) was used, considering a homogeneous structural matrix of the scale with indexes that make it possible to evaluate different aspects of the hypothesized model and its fit to the data: the goodness of fit index (GFI) measures the relative amount of variance explained by the model, an adjusted goodness of fit index (AGFI) is an indicator of the adjusted model, a non-normed fit index (NNFI) is an indicator of the relative model, the comparative fit index (CFI) indicates the degree of adjustment of the model and the parsimony goodness of fit index (PNFI) are an acceptable adjustment indicator model when their indexes exceeded a χ2 of 0.9 value and with root mean square error of approximation (RMSEA) values below 0.06 as the level of the total amount of error in the designed model [17].

Convergent validity was obtained from the statistical significance of the non-standardized critical saturation ratios of the items in each component, also considering a correlation greater than 0.5 as adequate [18]. Discriminant validity used the difference between the variance extracted for each component and the correlation with the square between the confirmed factors when the 95% confidence interval of the correlation between the two sub-scales did not hold the constant 1.0 [19]. Reliability was composed of the items using the proportion of the square of the sum of the standardized factor loadings over the total variance of the saturations, considering Cronbach’s alpha greater than 0.6 suitable in the design of new scales [20].

Interobserver reliability was calculated using the intraclass correlation coefficient (ICC) according to the sum of the scores obtained for each component on the scale recorded by the two observers, considering an ICC greater than or equal to 0.7 as the ideal value [18].

#### Score calculation of degrees of severity of postoperative complications in third molar surgery

The cut-off point for the degrees of postoperative complications severity after TMS was calculated by comparing the scores obtained from the scale with the degree of severity of complications recorded by the MFS or surgical resident (Table 3) using hierarchical segmentation analysis (classification and regression trees, CART) [21]. The method used determined cut-off scores, which, according to the severity of the complication, presented statistically significant differences via nonparametric tests (Kruskal-Wallis test and Dunn’s Method; *p* < 0.05).

The age of the patients, the MFS and the surgical resident, the number of third molars extracted and their degree of complexity as well as the scores on the scale were analyzed with descriptive statistics using percentages and measures of central tendency. The CFA was performed with SPSS v. 17 (SPSS Inc. Chicago, USA), CART with JMP v.8 and the structural equations model with LISREL v. 8.8 (Scientific Software International. Illinois, USA).

### Ethical considerations

The patients, surgeons and residents in the study participated voluntarily, after signing an informed consent. Both the study design and the work with patients were approved by the Bioethics and Research Committee of the Faculty of Medicine at the Universidad Austral de Chile (N° 2014/11/03).

## Results

During the period of study a total of 254 patients were attended for TMS in three Chilean hospitals, of which only 62 were selected according to the selection criteria and recording of complete data. Of these, the total number of third molars extracted was 104. The most frequently extracted teeth were the right mandibular third molar (40.3%) and right maxillary third molar (27.8%). Consistent with the degree of complexity, in 40.3% of the cases an osteotomy and odontosection were performed.

Nine patients (14.5%) had complications, classified according to the degree of severity: seven patients had a “mild complication” (five patients with alveolar osteitis and two with submucosal abscess) and two a “moderate complication” (both patients with subcutaneous abscess with alveolar osteitis and purulence). There were no cases of “severe complication”. In this evaluation, both group observers coincided in all cases with the classification provided in the degree of severity scale (Table 3).

The position indicators of the scores measured by each group of observers and the correlations of the eight proposed items appear in Table 4. In the calculation of the goodness-of-fit of the scale model, those items that showed no statistical significance (“trismus”, “erythema” and “suppuration”) were eliminated. Then, the model proposed grouping five items into two components. With these data, the structural equations model achieved a high degree of fit with a χ2 value of 0.53 and a RMSEA of 0.0001.

**Table 4:** Scores by observer. Significance of the saturations and criteria selection by convergent validity of the scale items

	Observer 1			Observer 2			Factor loadings of items in instrument	
Item	Median	Min	Max	Median	Min	Max	Standardized loadings	*p* value
Inflammation	2	1	5	3	1	7	0.608	< 0.01*
Erythema	1	1	2	1	1	2	0.307	0.033
Edema	1	1	5	1	1	5	0.768	< 0.01*
Pain	0	0	10	0	0	9	0.862	< 0.01*
Trismus	4	0	5	1	1	5	−0.032	0.818
Abscess	1	1	5	1	1	5	0.793	< 0.01*
Suppuration	1	1	10	1	1	10	0.376	0.012
Alveolar osteitis	1	1	10	1	1	10	0.664	< 0.01*

*Standardized loadings > 0.6 and *p* < 0.01 for selected items

The items “inflammation”, “edema” and “pain” were grouped into the first component; its standardized values are described with a composite Cronbach’s alpha of 0.79, an extracted variance of 56% and ICC interobserver reliability of 84.9%. The items “abscess” and “trismus” were grouped into the second component, their standardized values being described with a composite Cronbach’s alpha of 0.71, an extracted variance of 55% and ICC interobserver reliability of 87.9%, obtaining a final scale with a Cronbach’s alpha internal consistency of 0.71 and a discriminant validity of 0.50 (Table 5).

**Table 5:** Grouping of the items in the scale components, their factor loadings (Cronbach’s alpha), internal consistency, indexes of reliability, convergent and discriminant validity obtained by means of the exploratory factor analysis

Components	Composite Alpha	Extracted variance	Interobserver Reliability^a^	Item	Stand^b^	Var. Error	Factor loading^c^	Convergent validity^d^	Discriminant validity
Component 1	0.79	0.56	84.9	Inflammation	0.59	0.65	0.59	4.64	0.50
				Edema	0.74	0.45	0.74	6.11	
				Pain	0.89	0.21	0.81	6.68	
Component 2	0.71	0.55	87.9	Abscess	0.71	0.49	0.78	5.24	
				Alveolar osteitis	0.78	0.39	0.78	5.74	

^a^Intraclass correlation > 80%

^b^For selecting the item, the stand must be greater than the extracted variance of each component

^c^Cronbach’s alpha > 0.7

^d^Factor loading with statistical significance student’s t > 1.96

The model tested the data collected from empirical measures to determine the degree to which the data fit the model (Fit Index values > 0.9) [17] (Table 6). Therefore, the goodness-of-fit demonstrates the quality of the new scale model to replicate the results.

**Table 6:** The goodness-of-fit indexes according the hypothesized factorial model

χ2	p	df	χ2/df	GFI	RMSEA	NFI	NNFI	CFI	PNFI
3.15	0.53	4	0.78	0.98	0.0001	0.97	1.016	1	0.261

χ2: chi-square, *p*: *p*-value, *df*: degrees of freedom, *GFI*: goodness of fit index, *RMSEA*: root mean square error of approximation, *NFI*: normed fit index, *NNFI*: non-normed fit index, *CFI*: comparative fix index, *AGFI*: adjusted goodness-of-fit index, *NFI*: normed fit index, *PNFI*: parsimony goodness of fit index

Finally, the matrix obtained was able to discriminate the degree of severity through the sum of the scores on the scale used in three categories: “without or mild complication” (4–13 points), “moderate complication” (14–18 points) and “severe complication” (19–40 points). Only the category “without complications or mild complications” presented a statistically significant difference from the other two categories (*p* < 0.05) (Table 7).

**Table 7:** Surgical Complication Assessment Scale in Third Molar Surgery (SCATM)

Category	Item	Description	Score
Secondary complication	Inflammation	None	1
		Mild (intraoral and surgical area)	3
		Moderate (intraoral and extraoral)	5
		Severe (intraoral, extraoral and other regions of the head)	10
	Edema	None	1
		Intraoral	3
		Intraoral and extraoral	5
	Pain	Visual Analogue scale: How much pain does the patient feel?	0 to 10
Infection	Abscess	Absent	1
		Present	5
	Alveolar osteitis	Absent	1
		Present	10
		Total	4 to 40
		WITHOUT OR MILD COMPLICATION	4–13
		MODERATE COMPLICATION	14–18
		SEVERE COMPLICATION	19–40

## Discussion

A measurement instrument capable of recording and classifying the postoperative complications associated with TMS is presented. The scale stemming from the preliminary design [9] required a systematic search of the literature, selection of the suitable items in the opinion of experts meeting at the International Conference of Oral and Maxillofacial Surgeons (ICOMS 2011) and use of an exploratory factor analysis of the data.

### On the items and components of the scale

The first component named as “secondary complication” includes the intrinsic signs and symptoms resulting from an injury inherent to the hard and soft tissues in TMS, which vary depending on the characteristics of the patient and the surgery [1, 7, 22, 23]^.^ The second component, called “infection”, incorporates the semiological elements of an active infectious process that could aggravate the general condition in the presence of alveolar osteitis, considering the presence of microorganisms in the surgical wound [24, 25] associated with risk factors relevant to each patient such as the previous appearance of alveolar osteitis and poor oral hygiene [11].

### On the validity and reliability of the scale

The measurement model of the confirmatory factor analysis detected the items with the greatest validity and reliability on the scale with a percentage of explained variance and Cronbach’s alpha internal consistency of ≥0.70 [18, 20]. ICC reliability showed there was agreement about both constructs. Also, it was seen that the cut-offs were 13 and 18 points, which was consistent with the clinical diagnosis.

The data determined a highly homogenous structural matrix. The goodness-of-fit indicated a high degree of similarity between the information reproduced by the instrument and the diagnoses made by the observers. Additionally, the significance of χ2 corroborated that there were no statistically significant differences between the measurements obtained by the instrument and the diagnoses evaluated by MFS and surgical residents (Table 6). The hierarchical segmentation determined the correlation between the postoperative complication severity scores and the types of complications diagnosed by the observers. This exercise enabled the creation of an instrument from a theoretically significant and statistically acceptable model, affording the clinical researcher clarity in the classification of postoperative complications from TMS based on the total point score of the scale [19].

Despite the low complication rate reported here (14.5%), the confirmatory factor analysis and the structural equations model managed to clearly discriminate the complication levels proposed by the scale. The CART algorithm method used on this scale detected the optimal number of groups and their composition solely on the basis of the similarity between cases according to the scores obtained from the scale with the degree of severity of complications recorded by expert observers. These data contributed to discerning the complication rate, since it has been verified that the degree of inclusion in the bone, the presence of a pre-existing infection and pathology related to the position of the third molar are associated with the increased risk of complications in TMS [23, 26]. The purpose of this classification is so the clinician and oral surgeons can distinguish the levels of postoperative complications in cases similar to those observed by the experts, who described cases of alveolar osteitis, submucosal abscess as a “mild complication” and cases of subcutaneous abscess with purulent alveolar osteitis as a “moderate complication”. Although there were no cases of severe complications, in cases with an infectious involvement in other deep anatomical regions of the head or the airway, a higher score would be recorded, classified as a “severe complication”. The recording and evaluation of signs and symptoms enables a physical registry of the patients’ postoperative evaluation to be taken clearly, providing adequate patient care and an objective defense for surgeons to possibly prevent or defend against a negligence lawsuit in the event of an adverse outcome in a case of infection or major complication [27].

The limitation of this study was the variability of patients, surgeons and observers, which may cause measurement bias in the results section. Furthermore, the greatest difficulty was obtaining an adequate number of patients in each hospital who voluntarily participated in the study due to the high demand for care and the time required by the MFS and surgical trainee to complete the study protocol.

However, variability was controlled by taking the years of experience of the MFS, a single surgical protocol agreement [14] and the patient selection criteria into account. According to the aim of our study, the variability of the observations contributed to a greater variance of numerical data, allowing the scale to discriminate the level of complexity [15]. Despite the low rate of reported complications (14.5%), bias may be associated with the use in the use of this new instrument. However, the RMSEA value estimates the overall amount of error in this model, and here the value indicates an adequate fit of the model, which demonstrates the predictive capacity of the instrument. The score achieved in cases of complications was set according to the clinical diagnosis assigned by the experts. In addition, our results confirmed a new instrument with an appropriate internal consistency, reliability, convergent and discriminant validity (Table 4).

## Conclusions

In conclusion, a new measurement scale named “Surgical complication assessment scale in TMS” (SCATMS) is proposed for describing and quantifying the level of postoperative complications in TMS into none or mild, moderate and severe applied to patients in conditions similar to the sample used in our study. We propose future investigations to complement the instrument validation process in terms of its predictive value (predictive validity) and its behavior over time (test-retest). Additionally, the level of severity proposed will be analyzed with other populations or conditions and this instruments will be compared to more sensitive and specific instruments (e.g. biological markers) for infectious and inflammatory clinical status in the field of oral and maxillofacial surgery.

## References

[1] Susarla SM, Dodson TB (2004). Risk factors for third molar extraction difficulty. J Oral Maxillofac Surg.

[2] Haug RH, Perrott DH, Gonzalez ML, Talwar RM (2005). The American Association of Oral and Maxillofacial Surgeons age-Related Third Molar Study. J Oral Maxillofac Surg.

[3] Wiśniewska I, Slósarczyk A, Myśliwiec L, Sporniak-Tutak K (2009). Lincomycin applied to the alveolus on TCP carrier and its effect on wound healing after surgical extraction of a third molar. Ann Acad Med Stetin.

[4] Bui CH, Seldin EB, Dodson TB (2003). Types, frequencies, and risk factors for complications after third molar extraction. J Oral Maxillofac Surg.

[5] Larrazábal C, García B, Peñarrocha M, Peñarrocha M (2010). Influence of oral hygiene and smoking on pain and swelling after surgical extraction of impacted mandibular third molars. J Oral Maxillofac Surg.

[6] Sanchis Bielsa JM, Hernández-Bazán S, Peñarrocha Diago M (2008). Flap repositioning versus conventional suturing in third molar surgery. Med Oral Patol Oral Cir Bucal.

[7] Arteagoitia MI, Barbier L, Santamaría J, Santamaría G, Ramos E (2016). Efficacy of amoxicillin and amoxicillin/clavulanic acid in the prevention of infection and dry socket after third molar extraction. A systematic review and meta-analysis. Med Oral Patol Oral Cir Bucal.

[8] Norbeck JS (1985). What constitutes a publishable report of instrument development?. Nurs Res.

[9] Aravena PC, Astudillo P, Manterola C (2014). Design of a scale for measuring post-surgical complications in third molar surgery. Int J Oral Maxillofac Surg.

[10] Terwee CB, Bot SD, de Boer MR, van der Windt DA, Knol DL, Dekker J, Bouter LM, de Vet HC (2007). Quality criteria were proposed for measurement properties of health status questionnaires. J Clin Epidemiol.

[11] Blum IR (2002). Contemporary views on dry socket (alveolar osteitis): a clinical appraisal of standardization, aetiopathogenesis and management: a critical review. Int J Oral Maxillofac Surg.

[12] Bailey B, Daoust R, Doyon-Trottier E, Dauphin-Pierre S, Gravel J (2010). Validation and properties of the verbal numeric scale in children with acute pain. Pain.

[13] Speksnijder CM, van der Glas HW, van der Bilt A, van Es RJ, van der Rijt E, Koole R (2010). Oral function after oncological intervention in the oral cavity: a retrospective study. J Oral Maxillofac Surg.

[14] Hupp JR. Principles of Management of Impacted Teeth. In: Hupp JR, Ellis III E, Tucker MR, editors. Contemporary oral and maxillofacial surgery. 5th ed. St Louis: Mosby; 2008. p. 153.

[15] Hu LT, Bentler PM (1999). Cutoff criteria for fit indices in covariance structure analysis: conventional criteria versus new alternatives. Struct Equ Model Multidiscip J.

[16] Foguet JMB, Gallart GC (2000). Modelos de ecuaciones estructurales: modelos para el análisis de relaciones causales.

[17] Schreiber JB (2008). Core reporting practices in structural equation modeling. Res Social Adm Pharm.

[18] DeVon HA, Block ME, Moyle-Wright P, Ernst DM, Hayden SJ, Lazzara DJ, Savoy SM, Kostas-Polston E (2007). A psychometric toolbox for testing validity and reliability. J Nurs Scholarsh.

[19] Steiger JH (1980). Tests for comparing elements of a correlation matrix. Psychol Bull.

[20] Streiner DL, Norman GR. Selected of items. In: Streiner DL, editor. Health measurement scales. A practical guide to they development and use. 4th ed. New York: Oxford University Press; 2008. p. 194.

[21] Marshall RJ (2001). The use of classification and regression trees in clinical epidemiology. J Clin Epidemiol.

[22] de Santana-Santos T, de Souza-Santos JA, Martins-Filho PR, da Silva LC, de Oliveira E Silva ED, Gomes AC (2013). Prediction of postoperative facial swelling, pain and trismus following third molar surgery based on preoperative variables. Med Oral Patol Oral Cir Bucal.

[23] Popli G, Kiran DN, Iyer N, Sethi S, Bansal V, Bansal A (2015). Influence of Pederson score and its constitutional anatomical parameters to predict the postoperative morbidity after lower third molar removal: a prospective cohort study. American. J Oral Maxillofac Surg.

[24] Cardoso CL, Rodrigues MT, Ferreira Júnior O, Garlet GP, de Carvalho PS (2010). Clinical concepts of dry socket. J Oral Maxillofac Surg.

[25] Rodrigues MT, Cardoso CL, Carvalho PS, Cestari TM, Feres M, Garlet GP, Ferreira O (2011). Experimental alveolitis in rats: microbiological, acute phase response and histometric characterization of delayed alveolar healing. J Appl Oral Sci.

[26] Chuang SK, Perrott DH, Susarla SM, Dodson TB (2008). Risk factors for inflammatory complications following third molar surgery in adults. J Oral Maxillofac Surg.

[27] Holmes SM, Udey DK (2011). What are the lessons we can glean from a review of recent closed malpractice cases involving oral and maxillofacial infections?. Oral Maxillofac Surg Clin N Am.

